# Needs assessment for direct ophthalmoscopy training in neurology residency

**DOI:** 10.1186/s12909-024-05280-x

**Published:** 2024-03-27

**Authors:** Jasmeet Saroya, Noor Chahal, Alice Jiang, Douglas Pet, Nailyn Rasool, Mark Terrelonge, Madeline Yung

**Affiliations:** 1 UCSF Department of Ophthalmology Wayne and Gladys Valley Center for Vision, Box 4081, 490 Illinois Street, San Francisco, CA 94158 USA; 2 Department of Neurology, University of California, San Francisco, CA USA

**Keywords:** Fundoscopy, Education, Residency, Neurology

## Abstract

**Background:**

Assessment of the ocular fundus, traditionally by direct ophthalmoscopy (DO), is essential to evaluate many neurologic diseases. However, the status of DO training in neurology residencies is unknown. We conducted a needs assessment to determine current attitudes, curricula, and gaps in DO training.

**Methods:**

A survey was developed and administered to residents and program directors (PDs) at ACGME accredited neurology residencies in the United States. The survey assessed factors such as current DO curricula, perceived importance of DO, confidence of skills, and need for improvement. Data analysis was performed using the Mann Whitney U test and Fisher Exact Test.

**Results:**

Nineteen PDs (11.6%) and 74 (41.1%) residents responded to the survey. 97.1% of residents and 100.0% of PDs believe DO is an important skill to learn. 29.4% of PDs expected graduating residents to have completed > 10 supervised DO exams, while 0.0% of graduating fourth year residents reported doing so (*p* = 0.03). 35.7% of graduating residents had never correctly identified an abnormal finding on DO. The number of times residents practiced DO unsupervised correlated with increasing confidence in all components of the DO exam (*p* < 0.05). Residents who felt their program emphasized DO were more likely to perform DO at least once a week compared to residents who did not perceive program emphasis (61.9% vs. 35.0%, *p* = 0.02) and were more confident in DO (*p* < 0.05). 66.7% of residents and 42.1% of PDs were not satisfied with current levels of DO training. 96.7% of residents and 78.9% of PDs felt it was important to improve curriculum for DO training. Supervised practice and practice skills sessions were identified as the most helpful interventions to improve DO training.

**Conclusions:**

The vast majority of neurology PDs and residents believe DO is an important skill to learn, are unsatisfied with the current level of DO training, and advocate for improvement in DO curricula. Current DO curricula have limited formal didactic training and supervised practice. The bulk of DO learning occurs through unsupervised practice, which is influenced by motivational factors such as perceived residency emphasis on DO learning.

**Supplementary Information:**

The online version contains supplementary material available at 10.1186/s12909-024-05280-x.

## Background

Neurologic disorders often present with abnormalities in the ocular fundus, including but not limited to optic neuritis, ischemic optic neuropathy, or compressive optic neuropathy [1]. Many patients with neurologic complaints such as headache, eye pain, or vision loss require ophthalmoscopy to evaluate for fundoscopic abnormalities [1]. Thus, direct ophthalmoscopy (DO) is an important diagnostic tool that can play a major role in guiding vision or even life-saving interventions.

Direct ophthalmoscopy is widely recognized by authorities in undergraduate medical education as a core competency. The Association of American Medical Colleges (AAMC) expects medical students to be able to perform an ophthalmoscopic examination and have the capacity to describe findings [2, 3]. The Association of University Professors in Ophthalmology (AUPO) expects that medical students are capable of recognizing pathologies such as papilledema, glaucomatous cupping, and macular degeneration [4]. The International Council of Ophthalmology additionally expects students to recognize diabetic and hypertensive retinopathies [3, 4]. The standard neurology clerkship core curriculum developed by Gelb et al. lists fundoscopic examination as an essential skill for medical students on neurology clerkship [5]. This clerkship curriculum is endorsed by the Consortium of Neurology Clerkship Directors as well as the American Academy of Neurology (AAN), the American Neurological Association (ANA), and the Association of University Professors of Neurology (AUPN) [5].

The expectations regarding DO training during neurology residencies is less clear. Although the American Board of Psychiatry and Neurology’s (ABPN) core competencies outline and Accreditation Council for Graduate Medical Education’s (ACGME) neurology program requirements state that residents should have clinical and didactic experiences in neuro-ophthalmology and master the comprehensive neurologic examination, specific exam elements such as DO are not delineated [6].

The proficiency in DO for medical students and physicians in non-ophthalmology specialties has waned over the last several decades [7–9]. A needs assessment study done in South India demonstrated students were comfortable with visual acuity and pupillary reaction testing, but not with DO [10]. Meanwhile, a pilot study at Loyola University Medical Center in Chicago, IL showed low rates of DO by physicians in patients requiring fundoscopy in both inpatient and outpatient settings, with the Neurology service at 43% and other non-ophthalmology services scoring even lower rates [11]. The study reported that two thirds of the patients (66%) presenting with visual symptoms required evaluation with DO by Ophthalmology, which in some instances was crucial to making the final diagnosis.

Although evaluation of visual complaints and examination of the ocular fundus is inextricably entwined with the practice of neurology, and although DO is recognized as a core competency by governing bodies in undergraduate medical education, the training and use of DO has plummeted for medical students, neurologists, and other non-ophthalmology physicians [8]. There have been numerous efforts to address this gap in education for medical students including usage of DO simulations, supervised practice, or even e-learning support [12, 13]. Alternative and less technically difficult methods such as smartphone fundus photography and non-mydriatic fundus cameras have been proposed [14–18]. However, the requirements and training guidelines for fundoscopy in neurology residency are not clearly delineated by the governing bodies of graduate medical education or by professional neurology societies. It is unclear to what extent neurology residencies teach DO, or if neurology residents and program leadership even find value in learning DO.

This study represents the first needs assessment of DO and examination of the ocular fundus during neurology residency. It will identify the current attitudes toward DO by neurology program directors and residents in the United States. The study will assess current curriculum on DO, barriers to learning DO, and gaps in education in order to guide future interventions on DO education in neurology residencies.

## Methods

This study was approved by the University of California, San Francisco (UCSF) Institutional Review Board (IRB# 21-35509).

Needs assessment questionnaires were developed for neurology residents and neurology program directors (PDs) for adult neurology residency programs. The resident questionnaires assessed residents’ practice setting, subspecialty of interest, extent of formal and informal DO training, confidence of skills, perceived importance of DO, satisfaction with current curricula, and barriers to learning DO. The PD questionnaire was similar but assessed expected resident competence in DO rather than confidence of skills. Assessment of confidence in DO was modified from a previously validated questionnaire [19–22]. The questionnaires were reviewed by an Assistant Program Director for the UCSF Neurology residency (MT) and by the Division Chief of Neuro-Ophthalmology at UCSF (NR) for content. Questionnaires are available in the supplemental material.

The survey was active for 20 days, from 7/9/2022 to 7/28/2022. Informed consent for participation in the survey was distributed via the landing page for each questionnaire. Participants who consented to the study gave implied consent by proceeding to complete the questionnaire. Neurology residency program leadership contact information was obtained using the online Fellowship and Residency Electronic Interactive Database Access System (FREIDA) tool through the American Medical Association (AMA) website, and questionnaires were distributed to neurology PDs at 164 Accreditation Council for Graduate Medical Education accredited neurology residencies across the United States with available contact information. The PDs or residency program coordinators were asked to provide contact information for neurology residents for distribution of the resident questionnaire. Due to limited response rate by PDs and residency coordinators, only 180 neurology resident emails were received, and invitations were sent to these. The year of neurology residents was determined by the year of residency that they had most recently completed. For example, neurology residents who had recently completed their first year of neurology residency were designated as first year residents. Neurology residents who recently graduated were designated as fourth year residents. Residents who had graduated one or more years prior to survey distribution were excluded from analysis. Participation in the survey was anonymous, voluntary, and all survey questions were optional. A financial incentive of 5 was offered to the first 100 residents and $10 was offered to the first 50 program directors for survey completion.

Resident and PD survey data were analyzed to identify potentially significant relationships between variables. For questions regarding practice of DO, DO competence, and satisfaction with DO training, analyses were conducted primarily on second year (R2), third year (R3), and fourth year (R4) residents, as most first year (R1) neurology residents participate in an internal medicine intern year and do not receive neurology training. For questions regarding DO exposure in medical school and perceived importance of DO, all resident responses were analyzed. Residents were divided into “DO-heavy” and “Non-DO heavy” groups for sub-analysis based on their indicated subspecialty of interest. “DO heavy subspecialties” were defined as subspecialties likely to require DO in clinical practice including headache, neurocritical care, neuroimmunology, multiple sclerosis, and neuro-ophthalmology. For chi-square analysis of Likert scale responses, the two positive choices (somewhat confident, very confident) were used to denote those who were “more confident” and the other choices (neutral, somewhat unconfident, very unconfident) denoted those who were “less confident.” Categorical variables were analyzed with the Chi-Squared Test, and continuous variables were analyzed with the Mann-Whitney U Test and Fisher’s exact test with a p-value < 0.05.

## Results

A total of 19 (11.6%) PDs and 74 (41.1%) neurology residents responded to the survey from a total of 164 uniquely sent invitations to PDs and 180 invitations sent to residents. Graduating years for the residents that responded included: the class of 2022 (*n* = 14, 18.9%), 2023 (*n* = 20, 27.0%), 2024 (*n* = 27, 36.5%), and 2025 (*n* = 9, 12.2%). One resident response was excluded for not reporting a graduation year, and three resident responses were excluded due to reporting a graduation year of 2016 (*n* = 1) or 2026 (*n* = 2). Of total residents, 48.6% were male and 51.4% were female. Resident responses came from the following states: California (38.6%), Massachusetts (30.0%), Connecticut (8.6%), Pennsylvania (5.7%), Illinois (5.7%), Florida (4.3%), Louisiana (1.4%), New York (1.4%), Tennessee (1.4%), Maryland (1.4%), and Kentucky (1.4%). Residents reported exposure to the following practice settings: university hospital (91.4%), Veterans Affairs (VA) hospital (71.4%), county hospital (37.1%), and community-based hospital (24.3%). Residents also indicated intent to pursue the following subspecialities: headache (11.4%), movement disorders (8.6%), neurocritical care (8.6%), pediatrics (5.7%), neuroimmunology (5.7%), neurophysiology (4.3%), multiple sclerosis (4.3%), vascular (4.3%), epilepsy (2.9%), stroke (2.9%), behavioral (2.9%), neuro-ophthalmology (1.4%), neuromuscular (1.4%), neurohospitalist (1.4%), memory and aging (1.4%), neuro-oncology (1.4%), pediatric stroke (1.4%), pain (1.4%), or undecided/none (28.6%).

### Current curricula

The majority of residents reported exposure to DO in medical school, although only 18.6% of residents thought that their DO training in medical school was somewhat sufficient or very sufficient.

Of all PDs, 5.3% had a neurology residency program that required a neuro-ophthalmology rotation and 94.7% stated their programs had an elective neuro-ophthalmology rotation. 10.5% of PDs reported the residency did not include formal didactics on DO, 68.4% had 1–5 h, and 21.1% had > 5 h.

Residents also reported using various tools to perform a fundoscopic exam which included: direct ophthalmoscope (65.7%), PanOptic (87.1%), smartphone camera attachment (2.9%), fundus imaging (4.3%), and/or something else (1.4%). Only 8.2% of R2–R4s reported that they were trained on pupil dilation.

### Confidence/competence in direct ophthalmoscopy

**Fig. 1:**
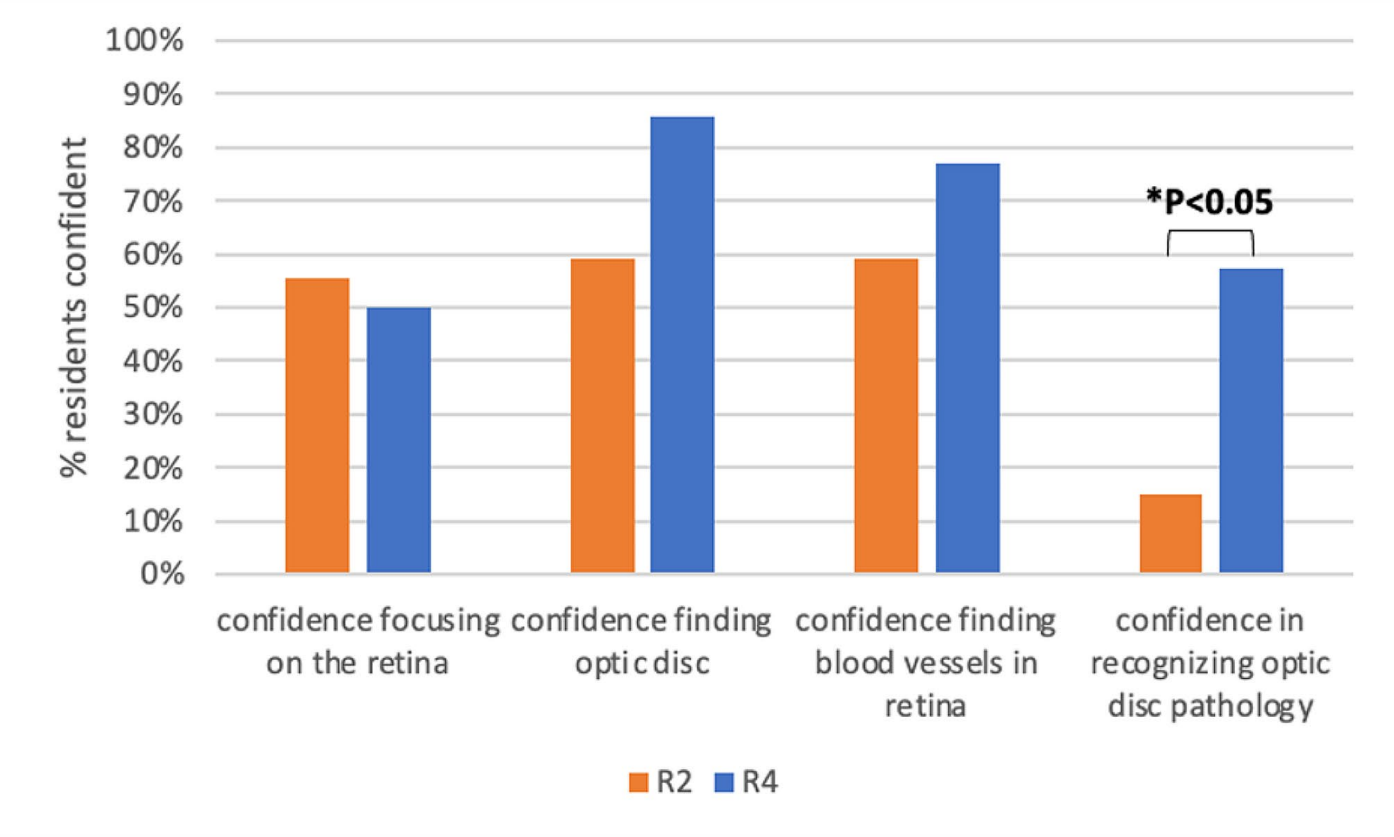
Resident confidence in direct ophthalmoscopy skills over training period. Percentage of second year neurology residents (R2, orange), compared to fourth year residents (R4, blue) who responded “somewhat confident” or “very confident” to different components of direct ophthalmoscopy

The percentage of residents responding that they were “somewhat confident” or “very confident” in the different components of DO were aggregated. For R2-R4s, 68.9% were confident in finding the optic disc, 32.8% were confident in recognizing optic disc pathology, 61.7% were confident in finding retinal blood vessels, and 55.7% were confident in focusing on the retina. Confidence in recognizing optic disc pathology significantly increased from 14.8% for R2s to 57.1% for R4s (*p* = 0.01), but not for the technical components of DO including focusing on retina, finding optic disc, or finding retinal blood vessels (Fig. 1).

**Fig. 2:**
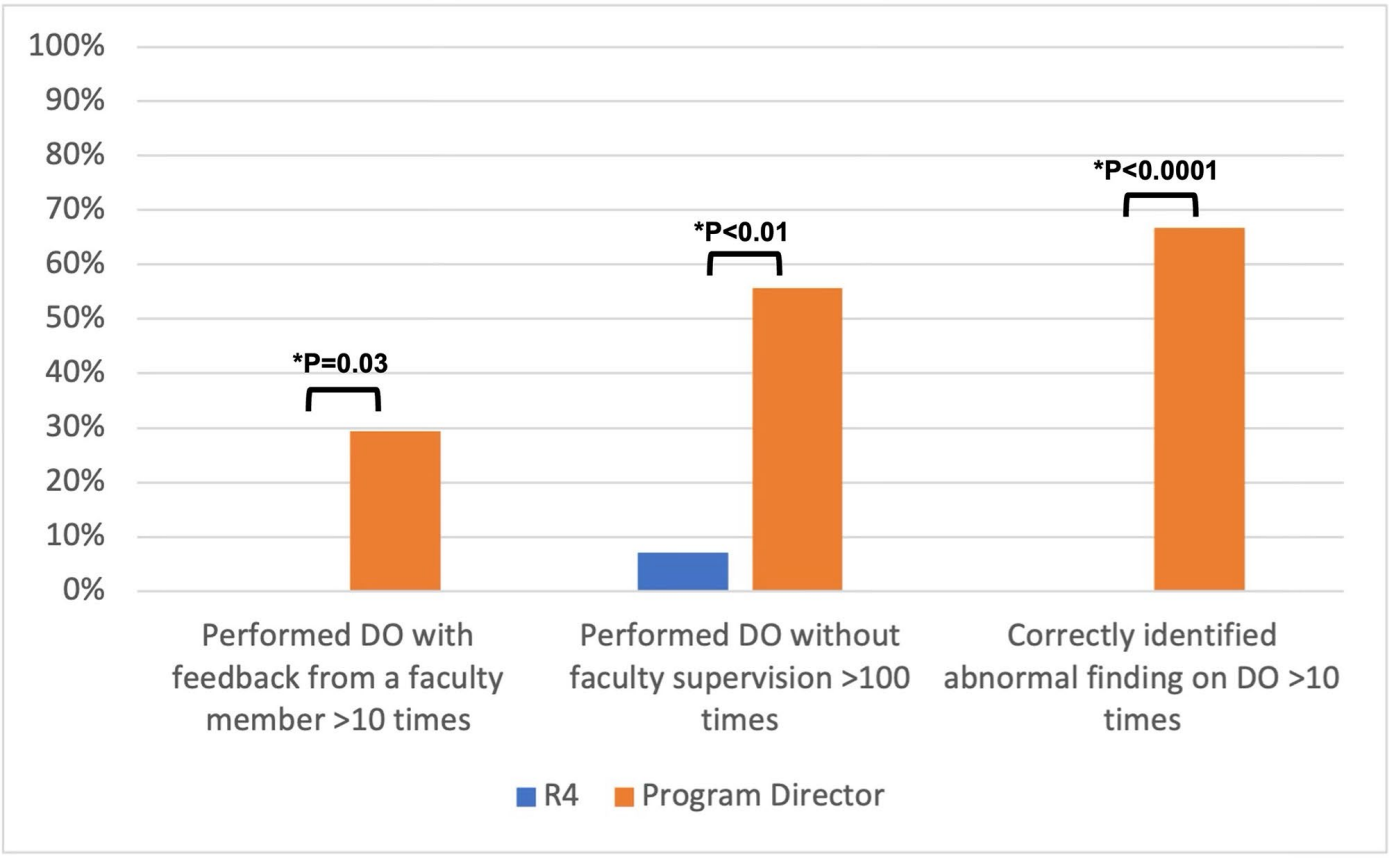
Program director expectations of resident performance of direct ophthalmoscopy (DO) skills compared to actual self-reported graduating fourth year resident (R4) performance of skills

Of the graduating fourth-year residents, 7.1% never performed DO with feedback from a faculty member, and 35.7% never correctly identified an abnormal finding on DO. In addition, PD expectations were compared with self-reported R4 performance of DO: 29.4% of PDs expected residents to perform DO > 10 times with feedback, compared to 0.0% of residents reporting that they had accomplished this (*p* = 0.03). Similarly, 55.6% of PDs expected > 100 times of unsupervised DO practice compared to 7.1% of residents who actually accomplished this (*p* < 0.01); and 66.7% of PDs expected > 10 times correctly identifying abnormal findings compared to 0.0% of residents who reported doing so (*p* < 0.0001) (Fig. 2).

**Fig. 3:**
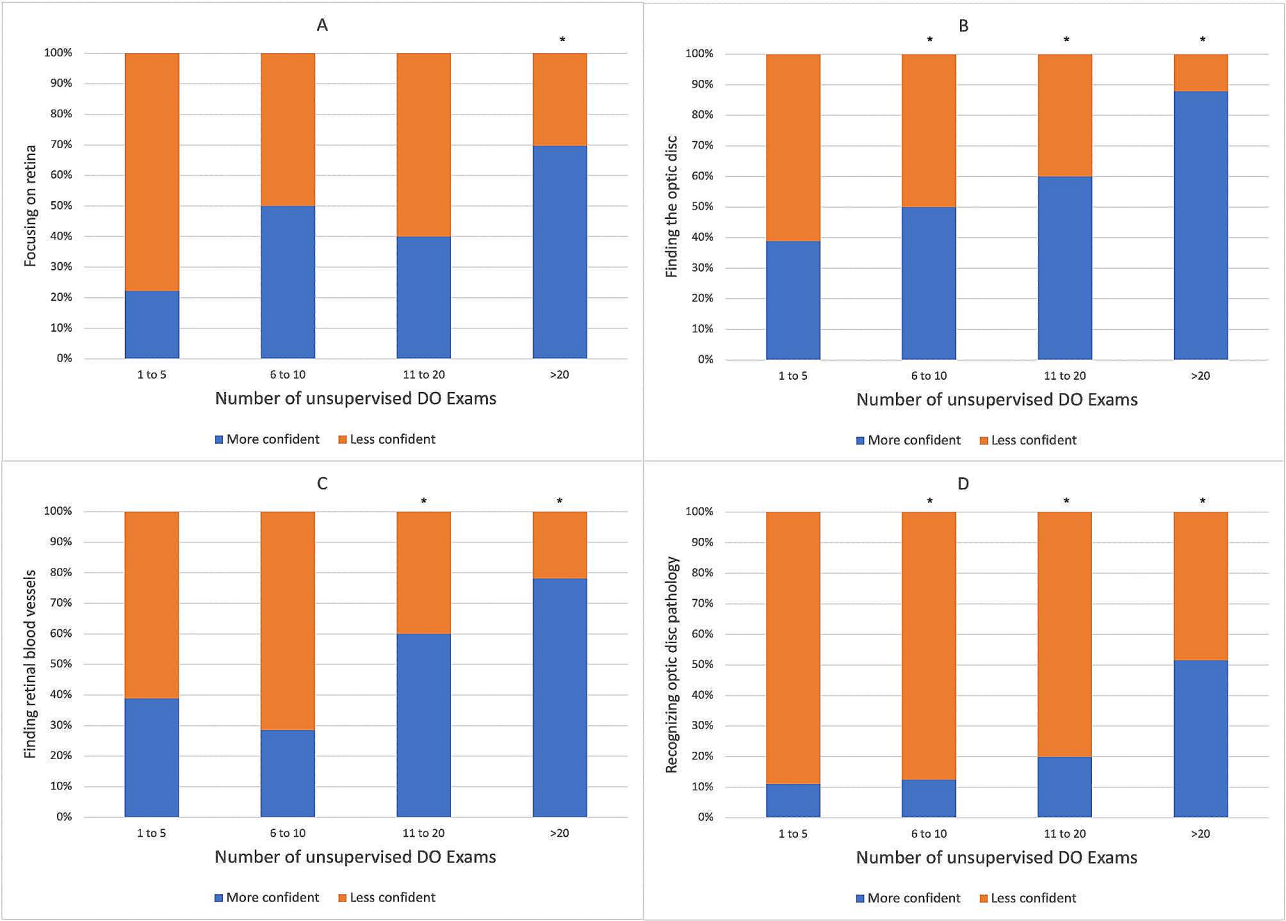
The number of unsupervised direct ophthalmoscopy (DO) exams performed correlated with increasing resident confidence in (**A**); focusing on the retina, (**B**); finding the optic disc, (**C**); finding retinal blood vessels, and (**D**); recognizing optic disc pathology. *A significant increase in confidence was detected, with *p* < 0.05

The number of times residents practiced DO unsupervised correlated with increasing confidence in performing the different components of DO. A significant increase in confidence emerged when DO was performed > 5 times for finding the optic disc (*p* = 0.02) and recognizing optic disc pathology (*p* = 0.04), > 10 times for finding retinal blood vessels (*p* = 0.01), and > 20 times for focusing on the retina (*p* = 0.01) (Fig. 3). 76.4% of residents who felt somewhat or very confident focusing on the retina intended to incorporate DO into practice after graduation, compared to 53.8% of residents who were unconfident (*p* = 0.03). On average, R4s performed unsupervised DO 44.4 times in residency, while R2s performed unsupervised DO 31.8 times (*p* = 0.03).

Residents that responded that they were very likely to incorporate DO into neurology practice were significantly more likely to perform DO frequently during residency, with 75.0% of them performing DO at least weekly compared to 29.2% for their colleagues (*p* = 0.01). There was no significant relationship for incorporating DO into clinical practice for residents pursuing DO-heavy subspecialties compared to those pursuing other fields (73.7 vs. 57.9%; *p* = 0.1). Interest in a DO-heavy subspecialty did not impact frequency or confidence in performing DO, satisfaction with DO education, or perceived importance of DO.

### Perception of direct ophthalmoscopy

A total of 97.1% of residents and 100.0% of program directors responded that they felt DO is an important skill to learn for neurology residents. However, only 36.2% of residents and 36.8% of program directors reported that their program placed emphasis on the fundoscopic exam. 21.7% of residents and 26.3% of program directors felt that AAN placed emphasis on the fundoscopic exam. For patients with increased intracranial pressure, residents believed that the following specialties should perform fundoscopy: ophthalmology (95.7%), neurology (95.7%), neurosurgery (21.4%), or optometry (15.7%). PDs had a similar response: ophthalmology (100.0%), neurology (84.2%), neurosurgery (10.5%), or optometry (10.5%).

Variables regarding attitudes and perceptions were analyzed for their impact on the intensity of DO practice by residents. 61.9% of residents who perceived that their residency emphasized DO were more likely to perform DO frequently (at least once a week), compared to 35.0% of residents in low emphasis residencies (*p* = 0.02). Similarly, 61.5% of R2-R4s who believed that DO was a “very important” skill to learn performed DO at least once a week compared to 13.6% of residents who did not (*p* < 0.001). Compared to R2-R4s who reported performing DO once a month or less, residents who reported performing DO at least once a week were more confident in all components of DO, including focusing on retina (*p* < 0.01), finding optic disc (*p* = 0.04), finding retinal blood vessels (*p* = 0.03), and recognizing optic disc pathology (*p* = 0.01). R2-R4s in high emphasis residencies were more confident in all components of the DO exam compared to low emphasis residencies: focusing on the retina (66.7% vs. 50.0%, *p* = 0.03), finding optic disc (85.7% vs. 59.0%, *p* = 0.02), finding retinal blood vessels (80.0% vs. 51.3%, *p* < 0.01), and recognizing optic disc pathology (42.9% vs. 27.5%, *p* = 0.02).

Residents who felt their program emphasized DO were much more likely to be satisfied with their DO education compared to residents who did not (42.8% vs. 5.0% respectively, *p* = 0.01).

### Gaps and potential interventions to DO training

A total of 65.6% of R2-R4s and 42.1% of PDs were not satisfied with the current level of DO training. All 96.7% of R2-R4s and 78.9% of PDs believed it was important to improve the curriculum for DO training during neurology residency.

**Fig. 4:**
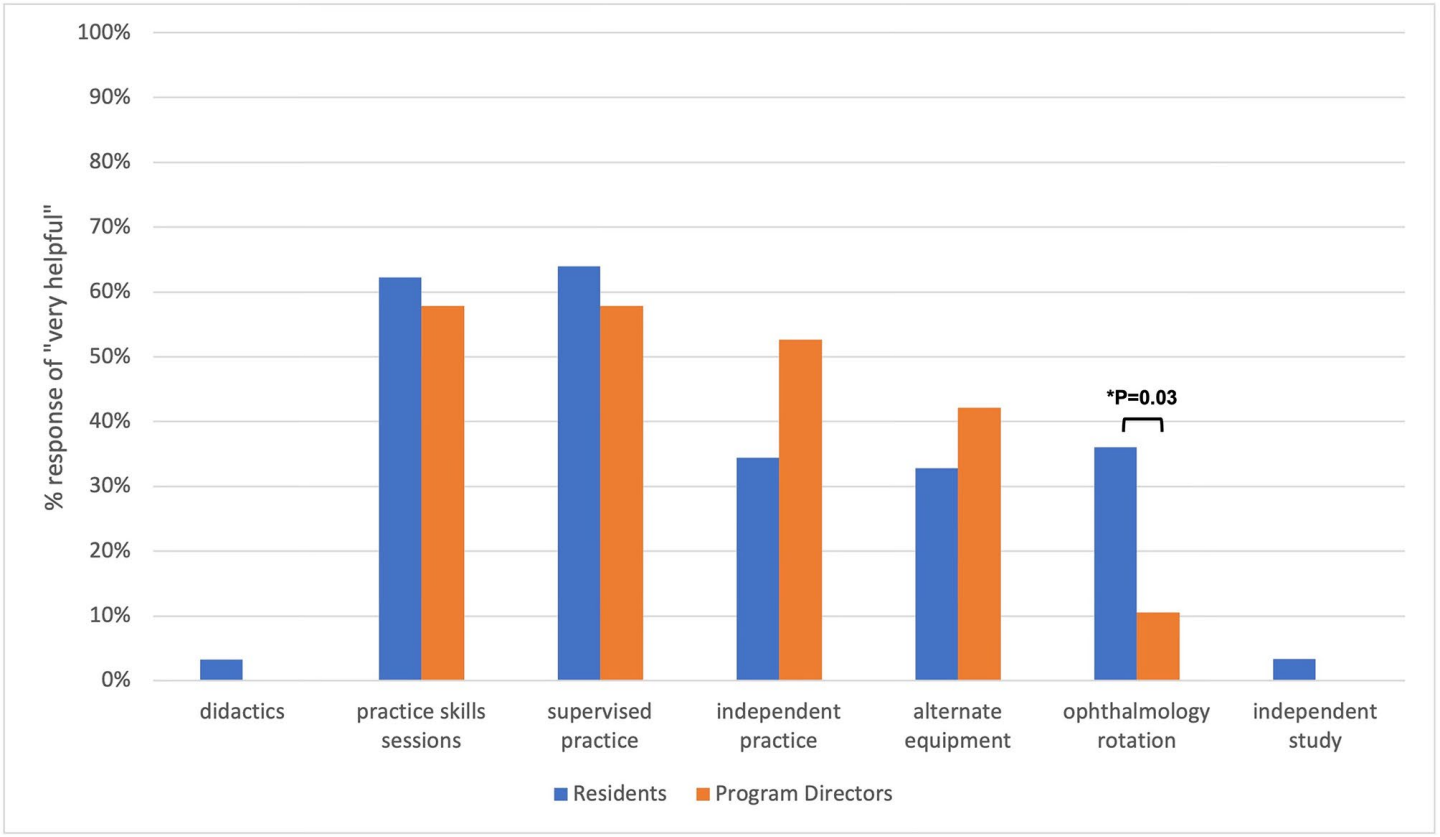
Perceived helpfulness of educational interventions for direct ophthalmoscopy training by neurology program directors and residents. Each category displays the percent of respondents selecting “very helpful.” Significant P-values are indicated with brackets

Survey participants were asked to rank various interventions by helpfulness in learning DO (Fig. 4). The most helpful interventions identified by both residents and PDs were practice skills sessions (85.5% and 89.5%, respectively) and supervised practice (91.2% and 89.5%, respectively). There was a significant difference in ranking ophthalmology rotations as an intervention, with 36.1% of residents ranking it as “most helpful” compared to 10.5% of PDs (*p* = 0.03).

**Fig. 5:**
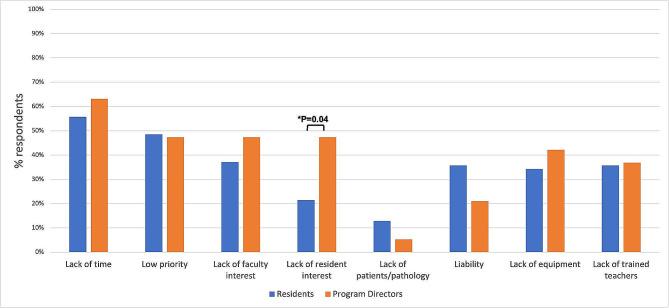
Barriers to direct ophthalmoscopy training for residents and program directors. Each category displays the percent of respondents selecting “somewhat” or “very significant.” Significant P-values are indicated with brackets

When asked to identify barriers to DO education, lack of time was the most commonly cited barrier, identified by over 50.0% of residents and PDs (Fig. 5). Low priority, lack of interest by faculty, lack of equipment, and lack of teachers trained in DO also received > 30.0% of responses. A higher percentage of PDs than residents perceived a lack of interest by residents as a barrier to DO education (47.4% vs. 21.4%, *p* = 0.04). Sub-analysis of residents who reported lack of interest by residents as a barrier did not find any significant associations with perceived value of DO or frequency of DO performance.

## Discussion

In this study, a needs analysis was performed on DO training in neurology residencies, with the aim to illuminate the current attitudes, perceptions, curricular development, barriers, and potential interventions to DO training. To the authors’ knowledge, this needs assessment represents the first such characterization of DO training on a national scale and provides unique data that can be used to inform future curriculum development.

Discrepancies in required competencies between medical school and neurology residency raise the question of whether fundoscopy still falls within the scope of neurology and whether neurology residents should be trained in direct ophthalmoscopy. While direct ophthalmoscopy is explicitly recognized as an essential competency by governing bodies in undergraduate medical education, this skill is not specifically mentioned in neurology residency competency guidelines published by the ABPN or ACGME [2, 3, 5, 6]. In addition, previous studies show low rates of DO performed by practicing neurologists [11]. This survey demonstrates a similar perception that professional societies such as AAN do not prioritize DO. However, the vast majority of survey respondents felt that DO was an important skill to learn, that neurologists should perform fundoscopy for common clinical scenarios, and that DO curriculum in neurology residencies should be improved. These findings indicate a consensus in the community of neurology education that DO falls within the scope of neurology and that neurology residents should be trained in DO. Further research is required to determine if this sentiment extends to the community of practicing neurologists and whether more specific guidance from governing bodies would improve resident competency in DO.

Due to the highly technical nature of DO, strategies to examine the ocular fundus with more user-friendly instruments have been explored. In the Fundus Photography vs. Ophthalmoscopy Trial Outcomes in the Emergency Department (FOTO-ED) study, Bruce et al. demonstrated high diagnostic accuracy, ease of access, and clinical utility of non-mydriatic fundus camera imaging in the emergency department by emergency department physicians and ancillary staff [23]. Meanwhile, smartphone fundoscopy using a portable attachment such as the D-eye adaptor (Padova, Italy) on an iPhone outperformed both non-mydriatic fundus cameras and DO in a direct comparison by medical students [17]. In this study, 4.3% of residents had used fundus imaging and 2.9% of residents had used smartphone imaging, compared to 65.7% who used DO and 87.1% who used the PanOptic. The low adoption rate of imaging technologies over the past decade may result in part from increased cost– non-mydriatic fundus cameras cost on average $3,000 - $5,000 and the D-eye attachment costs approximately $450 in addition to a smartphone– and in part variability between cameras and attachments which limit a standard educational approach. While fundus imaging is a promising avenue of fundoscopic examination, at this time its role in neurology residency education is limited by its low availability compared to DO. Even as fundus imaging grows in popularity, DO will likely still play a role in clinical evaluation, especially in low resource settings or when imaging is otherwise unavailable [24]. On the other hand, a higher proportion of residents reported using the PanOptic over the direct ophthalmoscope. Previous studies have demonstrated ease of use and learner preference for the PanOptic for DO, and modifying DO education sessions to focus on use of the PanOptic over the ophthalmoscope may enhance learning [25].

Despite clear delineation of DO educational guidelines by governing bodies of undergraduate medical education and exposure to DO during medical school for most neurology residents, the majority of survey respondents felt that medical school training was insufficient. Results of a pilot study in a medical school in New Mexico suggested that medical students’ direct ophthalmoscopy skills decrease over time without longitudinal skill reinforcement, indicating the need for ongoing training and evaluation to ensure students retain their skills in the long term [26]. There is a need for formalized DO training during graduate medical education in order to ensure competency.

There were large discrepancies between PD expectations in the number of supervised and unsupervised DO exams and the numbers reported by graduating residents. Further investigation into this discrepancy on an institutional level, for example by opening a dialogue between residency leadership and residents, or by implementing a case log, may identify gaps in DO education. Our findings indicate that the bulk of DO learning occurs at the discretion of the resident in the form of unsupervised practice, which correlated with increased confidence in all components of DO and the intention to incorporate DO into practice after graduation. Principles from self-regulated learning and motivation theory can be applied to optimize unsupervised DO practice. Training programs that promote self-regulated learning and intrinsic motivation [27] by emphasizing the importance of DO may be more effective in teaching it to their residents. In fact, residents who personally believed DO was an important skill to learn and who reported a high program emphasis in DO were more likely to perform DO at least once a week, were more confident in all components of DO, and were more satisfied with DO education. These results suggest that the culture surrounding DO set by residency program leadership impacts self-regulated learning and motivation to learn DO by residents.

Finding the optic disc and recognizing optic disc pathology appear to be the first components mastered, requiring only 5 examinations to achieve a significant increase in confidence. Focusing on the retina required the most examinations, > 20 to achieve an increase in confidence. This data suggests that when learning DO, residents progress from finding the optic disc and recognizing pathology, to finding the retinal blood vessels, to focusing on the retina. Focusing on the retina is the last technique to crystallize, and residents who indicated confidence in this final mechanic were more likely to incorporate DO into practice. Understanding the learning process for DO, in which residents sequentially master components of increasing difficulty, can help guide goal-setting for self-regulated learning and future curricular initiatives.

Confidence in recognizing optic disc pathology significantly increased from R2 to R4, but not for the technical components of DO such as focusing on retina, finding optic disc, or finding retinal blood vessels. The discrepancy in confidence between recognizing optic disc pathology compared to DO technique likely reflects learning achieved from viewing fundus photographs as opposed to identifying pathology during a live exam. Confidence does not necessarily reflect competence as early learners tend to overestimate competence [28]; R4s may be more competent, having performed a higher number of unsupervised DO exams relative to R2s despite reporting similar levels of confidence. Further study with objective assessments would be required to determine the actual competency of graduating neurology residents in DO, including the ability to recognize and identify optic disc pathology in a live patient as compared to a still image.

The reported barriers to learning DO align with previous studies demonstrating that insufficient time is a major limiting factor on medical education in scenarios with high clinical intensity [29]. Strategies to surmount this barrier include dedicating time specifically for the practice of DO, encouraging DO practice during less clinically demanding rotations, or taking advantage of time-flexible educational methods such as e-Learning, independent learning, or self-regulated learning [30]. Another common barrier encompasses a lack of educators - with a large portion of respondents reporting a lack of interest by faculty and a lack of teachers trained in DO. DO education initiatives for neurology faculty and their impact on neurology resident DO training is a potential avenue for curricular design that has yet to be investigated.

Interestingly, a higher proportion of residents compared to PDs ranked rotations through ophthalmology as a “most helpful” intervention for learning DO. Although DO is not routinely performed during ophthalmology clinic, ophthalmology rotations can offer concentrated and repeated opportunities to practice DO and exposure to ocular pathology. Offering an ophthalmology or neuro-ophthalmology rotation during neurology residency may be one way to further improve the DO curriculum.

Limitations for this study include a low survey response rate for both PDs and residents, which may impact the validity of study results and raises the question of selection bias, where PDs and residents who are more invested in DO may be more inclined to respond. Additionally, certain survey questions may be susceptible to recall bias, particularly those that asked residents to quantify their DO experiences during residency. With the small sample size, this study may be insufficiently powered to detect significant differences in the variables analyzed. Baseline data collected did show a relatively representative distribution of survey respondents by resident graduation year, practice settings, region of training, and subspeciality of interest. To protect resident anonymity, institutional identity was not collected, precluding assessment for interrater reliability within institutions. This factor limits the validity of subjective data including satisfaction with DO curricula and program emphasis on DO.

## Conclusion

In conclusion, the vast majority of neurology PDs and residents agree that DO is an important skill to learn and that DO training in neurology residency should be improved. Reports on the status of current curricula demonstrate limited formal didactic training and supervised practice. There is a low adoption of newer imaging technologies to examine the ocular fundus, but the PanOptic is more widely utilized over the direct ophthalmoscope. At this time, a significant proportion of learning DO falls on the resident independently practicing DO, an action that is influenced by motivational factors such as perceived residency emphasis and personal emphasis on DO education. This survey identifies several gaps in DO training, including insufficient time for learning, a discrepancy between PD expectations for number of exams and resident-reported numbers, low resident confidence in performing the technical components of DO, and an alarming proportion of graduating residents reporting that they had never identified an abnormal finding on DO. Potentially useful interventions were identified as practice sessions, supervised practice, and ophthalmology rotations. We hope that the data from this study will be useful in clarifying avenues for future investigation and curricular design of DO training, ultimately enhancing the ability of graduating neurology residents to feel confident in examining fundus findings in neurologic disease.

### Electronic supplementary material

Below is the link to the electronic supplementary material.

Supplementary Material 1

Supplementary Material 2

## Data Availability

The datasets used and/or analysed in the current study are available from the corresponding author on reasonable request.

## Abbreviations

DO: Direct ophthalmoscopy
FOTO-ED: Fundus Photography vs. Ophthalmoscopy Trial Outcomes in the Emergency Department
FREIDA: Fellowship and Residency Electronic Interactive Database Access System
ACGME: Accreditation Council for Graduate Medical Education
R#: # year neurology resident
AAMC: Association of American Medical Colleges
AUPO: Association of University Professors in Ophthalmology
AAN: American Academy of Neurology
ANA: American Neurological Association
AUPN: Association of University Professors of Neurology
ABPN: American Board of Psychiatry and Neurology
ACGME: Accreditation Council for Graduate Medical Education
PD: Program directors
UCSF: University of California, San Francisco
FREIDA: Fellowship and Residency Electronic Interactive Database Access System
AMA: American Medical Association
VA: Veterans Affairs
