# Supplementary material for: Needs assessment for direct ophthalmoscopy training in neurology residency
Source: BMC Med Educ. 2024 Mar 27;24:344. doi: 10.1186/s12909-024-05280-x (PMC10967108; doi:10.1186/s12909-024-05280-x)
Supplement: Supplementary file 1 — Supplementary Material 1 [file 12909_2024_5280_MOESM1_ESM.docx]

Program Director Questionnaire

Dear colleague,

We invite you to participate in an optional, anonymous survey conducted by the University of California, San Francisco (UCSF) Departments of Ophthalmology and Neurology.

**Due to the low response rate, the questionnaire compensation has been increased to a $10 Amazon gift card for the first 50 participants**, including those who have already responded.

Evaluation of fundoscopic examination education in neurology and neurosurgery residencies

This study examines the current landscape for training in direct ophthalmoscopy during neurology and neurosurgery residencies, via an UCSF Institutional Review Board (IRB) approved online survey to program directors and residents at ACGME-accredited programs. The results of this study will provide important background for the development of educational tools for the fundus exam.

This optional questionnaire will take less than 5 minutes to complete. You can stop the questionnaire at any time. All questions are optional, and you can submit a partially completed questionnaire. The questionnaire is anonymous, and no one will be able to link your answers back to you. Please do not include your name or other identifying information in your responses. You will also receive a separate email requesting resident contact information for the purposes of this study.

If you consent to the completion of this questionnaire, please click “NEXT” to begin the questionnaire.

Questions? Please contact Madeline Yung, MD at [madeline.yung@ucsf.edu](mailto:madeline.yung@ucsf.edu). If you have questions or concerns about your right as a research participant, you can call the UCSF IRB at 415-476-1814.

Directions:

Please answer the following questions pertaining to neurology residency. You may choose to leave any question unanswered or blank.

The term direct ophthalmoscopy is generally used to refer to examination of the ocular fundus, especially optic nerve pathology, which can be performed using a direct ophthalmoscope, pantoptic, smartphone camera, or any other examination method that provides adequate visualization of the fundus.

1. Age
2. Gender
3. State of residency (abbreviation, ie: CA for California)
4. Total number of neurology residents: free text
5. Number of full-time neurology department faculty
   1. 1-5
   2. 5-10
   3. 10-20
   4. 20+
6. Location of resident rotations: check all that apply
   1. University
   2. VA
   3. County
   4. Community-based
   5. Military
7. Please indicate the availability/requirement of a neuro-ophthalmology rotation:
   1. Required
   2. Elective
   3. Not available
8. How many hours of formal didactics are dedicated to direct ophthalmoscopy?
   1. 0 hours
   2. 1-5 hours
   3. 5-10 hours
   4. 10+ hours
9. How many hours of formal practice skills sessions (model eyes, standardized patients, etc) are dedicated to direct ophthalmoscopy?
   1. 0 hours
   2. 1-5 hours
   3. 5-10 hours
   4. 10+ hours
10. Please detail any additional formal curriculum on direct ophthalmoscopy, if any. Free text optional
11. Is there training for dilating a patient’s pupils for direct ophthalmoscopy?
    1. Yes
    2. No
12. For patients with suspected increased intracranial pressure, what service, if any, should perform fundoscopy? (check all that apply)
    1. Neurology
    2. Neurosurgery
    3. Ophthalmology
    4. Optometry
    5. None
13. In total during residency, about how many times should residents perform direct ophthalmoscopy with feedback from a faculty member (exam findings, technique, etc)?
    1. Free text number
14. In total during residency, about how many times should residents perform direct ophthalmoscopy without faculty supervision?
    1. Free text number
15. In total during residency, about how many times should residents correctly identify an abnormal fundoscopic finding?
    1. Free text number
16. Do you feel direct ophthalmoscopy overall is an important skill to learn for a neurology resident?
    1. Very important
    2. Somewhat important
    3. Neutral
    4. Somewhat not important
    5. Not important at all
17. Do you feel recognizing optic nerve pathology is an important skill to learn for a neurology resident?
    1. Very important
    2. Somewhat important
    3. Neutral
    4. Somewhat not important
    5. Not important at all
18. In your opinion, how competent are graduating residents with visualizing the optic nerve on fundoscopic examination?
    1. Very competent
    2. Somewhat competent
    3. Neutral
    4. Somewhat not competent
    5. Not competent at all
19. In your opinion, how competent are residents at recognizing optic nerve pathology?
    1. Very competent
    2. Somewhat competent
    3. Neutral
    4. Somewhat not competent
    5. Not competent at all
20. A competent graduating resident should be expected to visualize the optic nerve on what % of patients?
    1. Freetext number
21. Do you feel the residency program places emphasis on competency in the fundoscopic exam compared to other aspects of the general neurology curriculum?
    1. High emphasis
    2. Some emphasis
    3. Neutral
    4. Less emphasis
    5. No emphasis at all
22. Do you feel the American Academy of Neurology and other governing bodies place emphasis on competency in the fundoscopic exam compared to other aspects of the general neurology curriculum?
    1. High emphasis
    2. Some emphasis
    3. Neutral
    4. Less emphasis
    5. No emphasis at all
23. How satisfied are you with the current level of direct ophthalmoscopy training for residents?
    1. Very satisfied
    2. Somewhat satisfied
    3. Neutral
    4. Somewhat unsatisfied
    5. Unsatisfied
24. How important do you think it is to improve the curriculum for direct ophthalmoscopy during neurology residency?
    1. Not important
    2. Somewhat not important
    3. Neutral
    4. Somewhat important
    5. Very important
25. Please rank the following in terms of most helpful to least helpful for learning direct ophthalmoscopy
    1. Didactics
    2. Practice skills sessions
    3. Supervised practice on patients
    4. Independent practice on patients
    5. Use of alternative equipment (panoptic, smartphone attachment, etc)
    6. Rotations through neuro-ophthalmology / ophthalmology
    7. Independent study (reading)
26. What are the barriers to learning direct ophthalmoscopy during residency? Rank 1 to 5 from insignificant to very significant
    1. Lack of time
    2. Low priority
    3. Lack of interest by faculty
    4. Lack of interest by residents
    5. Lack of patients/pathology
    6. Liability (consult ophthalmology)
    7. Lack of equipment (direct ophthalmoscopes)
    8. Lack of teachers trained in fundoscopy
    9. Free text
27. Free text: What do you think would be the best way, if any, to improve direct ophthalmoscopy education?
28. Free text: Please include any thoughts or comments you have about direct ophthalmoscopy in general.
29. Please enter your email if you are interested in entering a raffle for a $XX Amazon gift card. Will delete if no funds available.
