# Supplementary material for: Needs assessment for direct ophthalmoscopy training in neurology residency
Source: BMC Med Educ. 2024 Mar 27;24:344. doi: 10.1186/s12909-024-05280-x (PMC10967108; doi:10.1186/s12909-024-05280-x)
Supplement: Supplementary file 2 — Supplementary Material 2 [file 12909_2024_5280_MOESM2_ESM.docx]

Fundoscopy questionnaire: graduating senior residents

Dear colleague,

We invite you to participate in an optional, anonymous survey conducted by the University of California, San Francisco (UCSF) Departments of Ophthalmology and Neurology. The first 100 participants have the option to enter their email to receive a $5 Amazon gift card. This email address is not linked to the survey response in any way.

**Evaluation of fundoscopic examination education in neurology and neurosurgery residencies**

This study examines the current landscape for training in direct ophthalmoscopy during neurology and neurosurgery residencies, via an UCSF Institutional Review Board (IRB) approved online survey to program directors and residents at ACGME-accredited programs. The results of this study will provide important background for the development of educational tools for the fundus exam.

This optional questionnaire will take about 7 minutes to complete. You can stop the questionnaire at any time. All questions are optional, and you can submit a partially completed questionnaire. The questionnaire is anonymous, and no one will be able to link your answers back to you. Please do not include your name or other identifying information in your responses.

If you consent to the completion of this questionnaire, please click “NEXT” to begin the questionnaire.

Questions? Please contact Madeline Yung, MD at [madeline.yung@ucsf.edu](mailto:madeline.yung@ucsf.edu). If you have questions or concerns about your right as a research participant, you can call the UCSF IRB at 415-476-1814.

Directions:

Please answer the following questions pertaining to neurology residency. You may choose to leave any question unanswered or blank.

The term direct ophthalmoscopy is generally used to refer to examination of the ocular fundus, especially optic nerve pathology, which can be performed using a direct ophthalmoscope, pantoptic, smartphone camera, or any other examination method that provides adequate visualization of the fundus.

1. Age
2. Gender
3. What year will you graduate neurology residency?
4. State of residency
5. Location of rotations: check all that apply
   1. University
   2. VA
   3. County
   4. Community-based
   5. Military
6. What subspecialty, if any, are you pursuing (if none, write none)? Free text
7. Please indicate the availability/requirement of a neuro-ophthalmology rotation Required
   1. Elective
   2. Not available
8. How many hours of formal didactics on direct ophthalmoscopy have you attended during residency?
   1. 0 hours
   2. 1-5 hours
   3. 5-10 hours
   4. 10+ hours
9. How many hours of formal practice skills sessions (model eyes, standardized patients, etc) for direct ophthalmoscopy have you attended during residency?
   1. 0 hours
   2. 1-5 hours
   3. 5-10 hours
   4. 10+ hours
10. Were you trained on dilating a patient’s pupils for direct ophthalmoscopy?
    1. Yes
    2. No
11. Have you performed a dilated direct ophthalmoscopy exam?
    1. Yes
    2. No
12. What fundoscopy equipment do you generally use for an exam (check all that apply)?
    1. Direct ophthalmoscope
    2. Panoptic
    3. Smartphone camera attachment
    4. Fundus imaging
    5. Other: free text
13. How many faculty members have you been on rotation with who use direct ophthalmoscopy confidently as part of their practice? Freetext
14. What % of time during residency is spent training with faculty members who regularly perform direct ophthalmoscopy as part of their practice?
    1. 0%
    2. 1-10%
    3. 11-30%
    4. 30-50%
    5. >50%
15. For patients with suspected increased intracranial pressure, what service, if any, should perform fundoscopy? (check all that apply)
    1. Neurology
    2. Neurosurgery
    3. Ophthalmology
    4. Optometry
    5. None
16. How many hours of formal or informal training did you receive in medical school for direct ophthalmoscopy?
    1. 0 hours
    2. 1-5 hours
    3. 5-10 hours
    4. 10-25 hours
    5. 25+ hours
17. What was the format of training? (check all that apply)
    1. Lecture
    2. Skills session model eyes
    3. Skills session standardized patients/other students
    4. Skills session on simulator
    5. Supervised practice on a patient
    6. Unsupervised practice on a patient
    7. Free text
18. Was the training from medical school sufficient to feel confident in direct ophthalmoscopy as a resident?
    1. Not sufficient
    2. Somewhat insufficient
    3. Neutral
    4. Somewhat sufficient
    5. Sufficient
19. In total during residency, about how many times have you performed direct ophthalmoscopy with feedback from a faculty member? (exam findings, technique, etc) Freetext
20. In total during residency, about how many times have you performed direct ophthalmoscopy without faculty supervision? freetext
21. In total during residency, about how many times have you correctly identified an abnormal finding on direct ophthalmoscopy during residency? freetext
22. In total during residency, about how many times have you observed a senior resident, fellow, or attending perform direct ophthalmoscopy?
23. How often do you perform direct ophthalmoscopy?
    1. At least once a day
    2. At least once a week
    3. At least once a month
    4. Rarely
24. Do you feel direct ophthalmoscopy is an important skill to learn for a neurology resident?
    1. Very important
    2. Somewhat important
    3. Neutral
    4. Somewhat not important
    5. Not important at all
25. Do you feel recognizing optic nerve pathology is an important skill to learn for a neurology resident?
    1. Very important
    2. Somewhat important
    3. Neutral
    4. Somewhat not important
    5. Not important at all
26. Do you feel the residency program places emphasis on competency in the fundoscopic exam compared to other aspects of the general neurology curriculum?
    1. High emphasis
    2. Some emphasis
    3. Neutral
    4. Less emphasis
    5. No emphasis at all
27. Do you feel the American Academy of Neurology and other governing bodies place emphasis on competency in the fundoscopic exam compared to other aspects of the general neurology curriculum?
    1. High emphasis
    2. Some emphasis
    3. Neutral
    4. Less emphasis
    5. No emphasis at all
28. Do you feel confident with focusing the ophthalmoscope onto the retina?
    1. Very confident
    2. Somewhat confident
    3. Neutral
    4. Somewhat unconfident
    5. Very confident
29. Do you feel confident with finding the optic disc?
    1. Very confident
    2. Somewhat confident
    3. Neutral
    4. Somewhat unconfident
    5. Very confident
30. Do you feel confident finding the blood vessels in the retina?
    1. Very confident
    2. Somewhat confident
    3. Neutral
    4. Somewhat unconfident
    5. Very confident
31. Do you feel confident in recognizing optic disc pathology?
    1. Very confident
    2. Somewhat confident
    3. Neutral
    4. Somewhat unconfident
    5. Not confident
32. In your best estimate, in what % of patients can you successfully visualize the optic nerve on fundoscopic exam?
    1. Freetext number
33. How likely is it that you will incorporate direct ophthalmoscopy into your neurology practice after graduation? Yes/no
34. How satisfied are you with the current level of direct ophthalmoscopy training for residents?
    1. Very satisfied
    2. Somewhat satisfied
    3. Neutral
    4. Somewhat unsatisfied
    5. Unsatisfied
35. How important do you think it is to improve the curriculum for direct ophthalmoscopy during neurology residency?
    1. Not important
    2. Somewhat not important
    3. Neutral
    4. Somewhat important
    5. Very important
36. Please rank the following in terms of most helpful to least helpful for learning direct ophthalmoscopy
    1. Didactics
    2. Practice skills sessions
    3. Supervised practice on patients
    4. Independent practice on patients
    5. Use of alternative equipment (panoptic, smartphone attachment, etc)
    6. Independent study (reading)
37. What are the barriers to learning direct ophthalmoscopy during residency? Rank 1 to 5 from insignificant to very significant
    1. Lack of time
    2. Low priority
    3. Liability (consult ophthalmology)
    4. Lack of interest by faculty
    5. Lack of faculty who are trained in fundoscopy
    6. Lack of interest by residents
    7. Lack of patients/pathology
    8. Lack of equipment (direct ophthalmoscopes)
    9. Free text
38. Free text: What do you think would be the best way, if any, to improve direct ophthalmoscopy education?
39. Please include any thoughts or comments you have about fundoscopy in general.
40. Please enter your email if you are interested in entering a raffle for a $XX Amazon gift card. Will delete if no funds available.
